# How Social Isolation Exacerbates Unmet Care Needs in Older Adults With Dementia

**DOI:** 10.1093/geroni/igaf122.582

**Published:** 2025-12-31

**Authors:** Zhuoer Lin, Zhiyong Lin

**Affiliations:** University of Illinois Chicago, Chicago, Illinois, United States; University of Texas at San Antonio, San Antonio, Texas, United States

## Abstract

Older adults living with dementia (PLWD) have substantial care needs, yet many face significant barriers to accessing adequate support. Social isolation is particularly concerning, as it restricts access to care networks and is associated with poorer health outcomes, potentially exacerbating unmet care needs among PLWD. While social isolation is recognized as a modifiable risk factor for dementia, its impact on unmet care needs remains insufficiently explored. This study examines the association between social isolation and unmet care needs among PLWD aged 65 and older using longitudinal data from the National Health and Aging Trends Study (NHATS, 2011–2018, N = 5,690). By classifying individuals based on their levels of social isolation, care needs, and care network structures, we assess how these factors intersect. Mixed-effects negative binomial regression models reveal that socially isolated PLWD, particularly those experiencing severe isolation, have significantly higher rates of unmet care needs compared to their socially integrated counterparts. While disparities in access to supportive networks and differences in care needs contribute to this pattern, severe social isolation continues to be strongly associated with higher unmet care needs even after accounting for these factors. These findings highlight the urgent need for targeted interventions to enhance social and care networks for PLWD, especially those who are most isolated. Reducing social isolation could be key to addressing unmet care needs, improving health outcomes, and enhancing overall well-being among PLWD. Policymakers and healthcare providers should prioritize efforts to mitigate social isolation as part of a comprehensive strategy to improve dementia care.

